# Prediction of activity cliffs on the basis of images using convolutional neural networks

**DOI:** 10.1007/s10822-021-00380-y

**Published:** 2021-03-19

**Authors:** Javed Iqbal, Martin Vogt, Jürgen Bajorath

**Affiliations:** Department of Life Science Informatics, B-IT, LIMES Program Unit Chemical Biology and Medicinal Chemistry, Rheinische Friedrich-Wilhelms-Universität, Friedrich-Hirzebruch-Allee 6, 53115 Bonn, Germany

**Keywords:** Activity cliffs, Matched molecular pairs, Image analysis, Convolutional neural networks, Convolutional feature visualization

## Abstract

An activity cliff (AC) is formed by a pair of structurally similar compounds with a large difference in potency. Accordingly, ACs reveal structure–activity relationship (SAR) discontinuity and provide SAR information for compound optimization. Herein, we have investigated the question if ACs could be predicted from image data. Therefore, pairs of structural analogs were extracted from different compound activity classes that formed or did not form ACs. From these compound pairs, consistently formatted images were generated. Image sets were used to train and test convolutional neural network (CNN) models to systematically distinguish between ACs and non-ACs. The CNN models were found to predict ACs with overall high accuracy, as assessed using alternative performance measures, hence establishing proof-of-principle. Moreover, gradient weights from convolutional layers were mapped to test compounds and identified characteristic structural features that contributed to successful predictions. Weight-based feature visualization revealed the ability of CNN models to learn chemistry from images at a high level of resolution and aided in the interpretation of model decisions with intrinsic black box character.

## Introduction

In recent years, convolutional neural networks (CNNs) have gained increasing attention in chemical informatics and pharmaceutical research. For example, two-dimensional (2D) images of molecular graphs [1–5] and three-dimensional (3D) images of activity landscapes [6] have been used for deriving CNN models and extracting specific features from image data. For example, the Inception-ResNet v2 architecture was used to train CNN models on images from a large data set comprising 1.7 million compounds and predict physicochemical properties such as logP [1]. In addition, quantitative property predictions on the basis of compound images were reported using Chemception [2] and ChemNet [3]. Furthermore, the Toxic Colors approach [4] added atom labels, colored dots, and partial charge maps to image representations for compound toxicity predictions while Kekulescope [5] only used Kekulé structures as input for compound potency and cell line toxicity predictions. Taken together, these investigations have indicated the potential of various CNN architectures to extract specified molecular features from 2D image representations and use these features for property predictions. Different from molecular structure-based approaches, 3D images of activity landscape variants were used for feature extraction and classification of landscape models according to structure–activity relationship (SAR) characteristics of the corresponding compound data sets [6].

While CNNs have thus far mostly been trained on 2D compound images, to our knowledge, they have not been used to process images of pairs of closely related compounds and predict differences in properties at the level of pairs. Activity cliffs (ACs) represent a prominent paradigm for compound pair-encoded property differences [7]. ACs are defined as pairs or groups of similar compounds or structural analogs with large differences in activity (potency) [7, 8]. Accordingly, ACs embody the pinnacle of SAR discontinuity, i.e., small chemical modifications leading to large potency alterations, and are a major source of SAR information [8]. An elegant formalism for the systematic identification of pairs of structural analogs is the matched molecular pair (MMP) concept and its algorithmic implementation [9]. An MMP is defined as a pair of compounds that share a common core structure and are only distinguished by a chemical modification at a single site (termed a chemical transformation) [9]. As such, MMPs are well suited for representing ACs, which has led to the introduction of MMP-cliffs [10]. An MMP-cliff is defined as an MMP formed by two compounds that are active against the same target and have a statistically significant difference in potency [10].

As a consistent molecular representation, MMP-cliffs have been used for predicting ACs at different levels. First, MMP-cliffs have been systematically distinguished from MMPs with only small or no potency differences using support vector machine classification on the basis of fingerprint representations and specialized compound pair-based kernel functions [11]. Subsequently, MMP-cliffs have also been successfully predicted in a methodologically simpler manner applying the condensed graph of reaction formalism [12]. In addition, potency differences encoded by MMPs have been quantitatively predicted using support vector regression [13]. To aid in the interpretation of machine learning models, fingerprint features determining correct AC predictions have been mapped back to the original compounds to delineate critically important substructures distinguishing MMP-cliffs from other MMPs [11].

Herein, we have attempted to predict MMP-cliffs from image data using CNNs. In addition to assessing classification performance, we have made use of recent developments in convolutional layer visualization [14–17] to identify and display key features contributing to correct AC predictions. Our proof-of-concept investigation further extends the current spectrum of molecular image-based modeling in chemical informatics.

## Material and methods

### Compound activity classes

From ChEMBL (version 26) [18], three compound activity classes with available high-confidence activity data were extracted. Compounds were tested against single human targets in direct interaction assays at highest assay confidence (ChEMBL confidence score 9). As potency measurements, assay-independent equilibrium constants (pK_i_ values) were required. Multiple measurements for the same compound were averaged, provided all values fell within the same order of magnitude; otherwise, the compound was disregarded. Table 1 reports the targets and composition of these activity classes.

**Table 1:** Activity classes

Target name	ChEMBL target ID	MMP-cliffs			Non-AC MMPs		
		MMPs	Unique cores	Unique substituents	MMPs	Unique cores	Unique substituents
Thrombin	204	456	61	168	3595	554	567
Tyrosine kinase Abl	1862	1122	37	251	6143	322	419
Mu opioid receptor	233	466	114	286	9712	1230	959

For each activity class, the total number of compounds, MMP-cliffs, non-AC MMPs, unique core structures, and substituents are reported

### Matched molecular pairs and activity cliffs

For activity classes, all possible MMPs were generated by systematically fragmenting individual exocyclic single bonds and sampling core structures and substituents in index tables [9]. For substituents, size restrictions were applied to limit MMP formation to typically observed structural analogs [10]. Accordingly, a substituent was permitted to contain at most 13 non-hydrogen atoms and the core had to be at least twice as large as the substituent. Additionally, for MMP compounds, the maximum difference in non-hydrogen atoms between the substituents was set to eight, yielding transformation size-restricted MMPs [10].

An MMP qualified as an MMP-cliff if the two structural analogs had an at least 100-fold difference in potency (ΔpK_i_ ≥ 2.0) [10]. To avoid potency difference-dependent boundary effects in AC prediction, compounds forming a non-AC MMP were restricted to an at most tenfold difference in potency. Furthermore, to balance structural heterogeneity of large activity classes originating from different sources, MMPs were only retained if their compounds and core structures were found in multiple MMPs. Table 1 reports MMP and MMP-cliff statistics for the activity classes.

### Molecular image representations

**Fig. 1:**
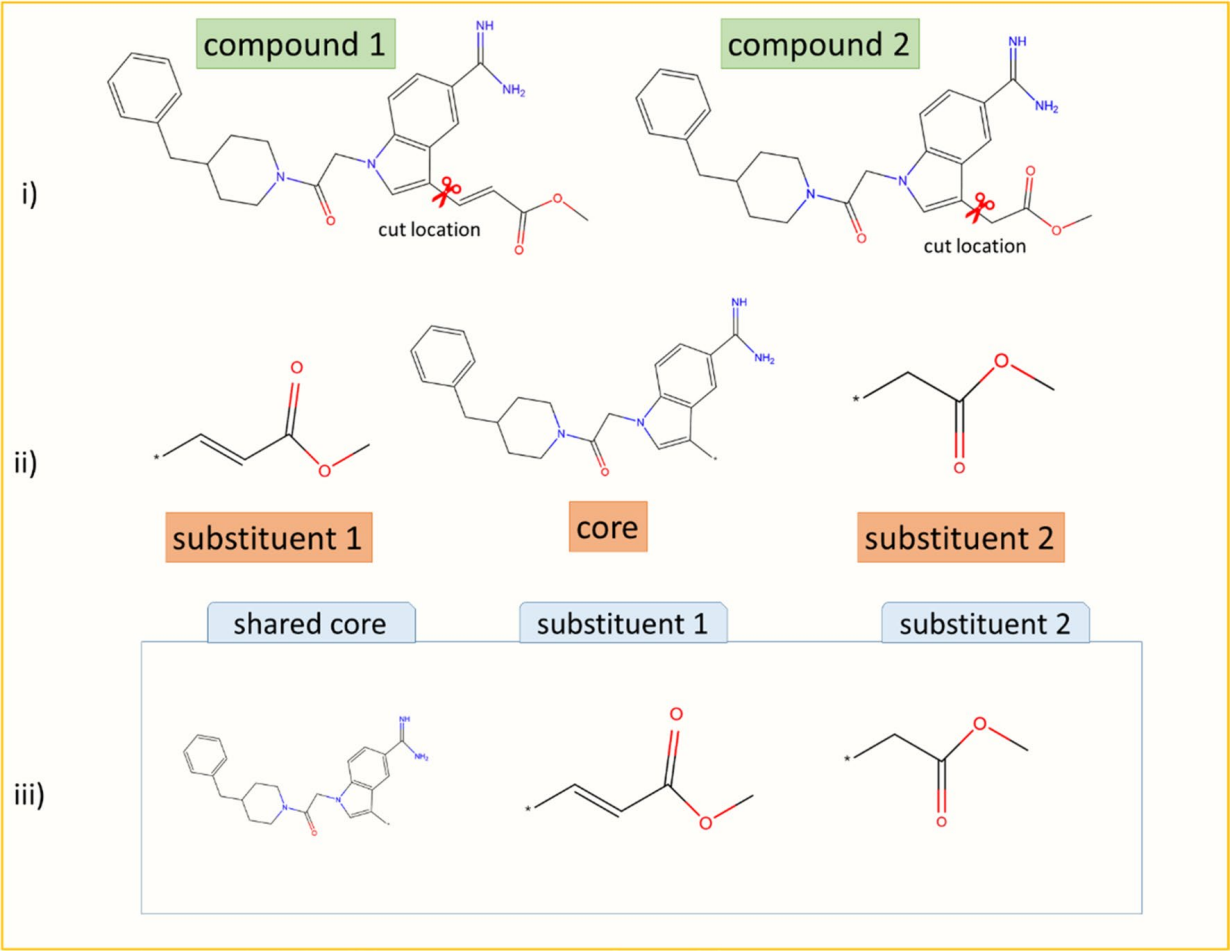
Generation of MMP images. **i** Two compounds forming an MMP are shown. **ii** The common core and the two substituents defining the chemical transformation are displayed. **iii** Separate core and substituent images are horizontally concatenated yielding a single image

Each MMP core and the associated substituents were treated as separate molecular objects using the RDKit application programming interface (API) [19]. For each unique core and substituent, high-resolution portable network graphics (PNG) compound images with 500 × 500 pixels were generated using the RDkit Chem.Draw package (version 2020.03.5) [19]. In images, substituent attachment sites were replaced with an asterisk symbol. To represent an MMP core and the two substituents defining the transformation in combined form, core and substituent images were resized to 300 × 300 pixels and then horizontally concatenated in a single image of dimensions 300 × 900 × 3 (height × width × color-channels). Figure 1 illustrates MMP image generation. The pixel values of all image matrices were converted into 32-bit floating point format and normalized. Images were processed using openCV (version 4.4.0) [20–22].

### Convolutional neural network architecture

**Fig. 2:**
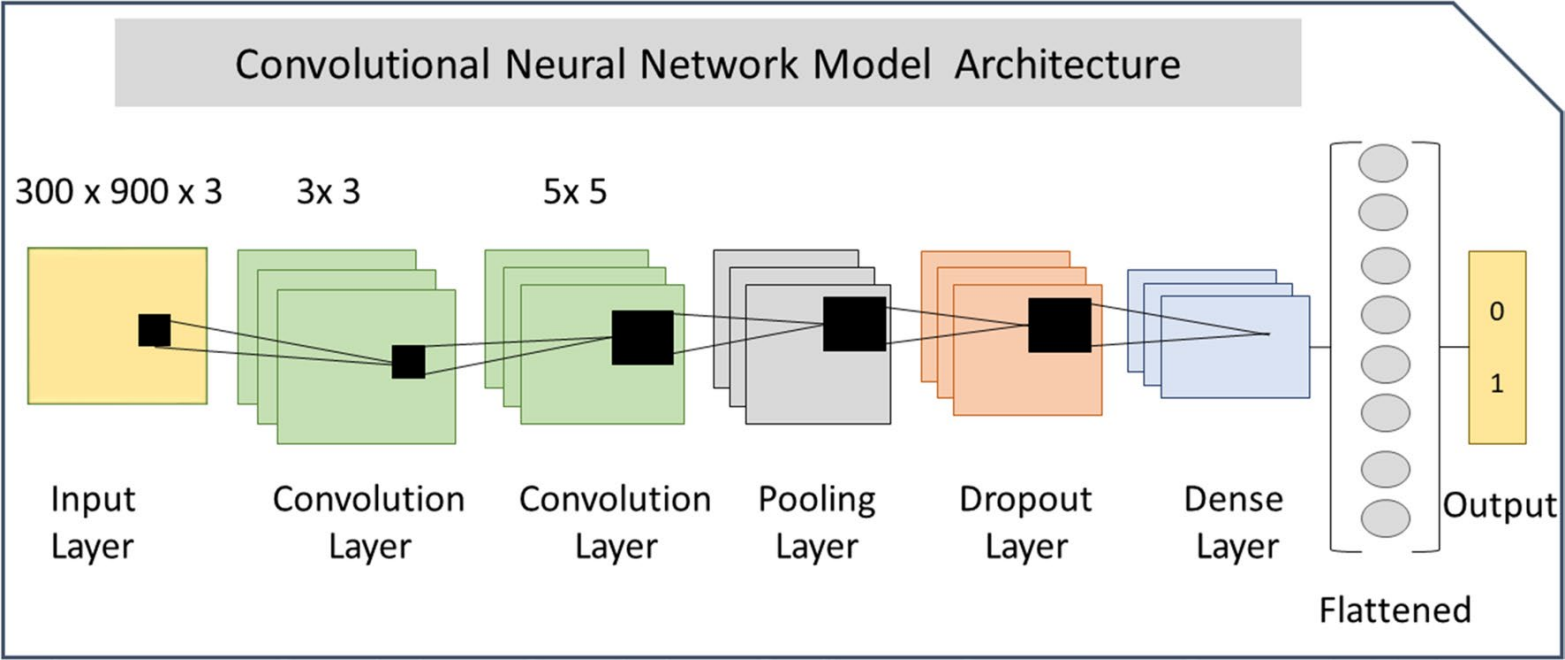
The CNN model architecture used for MMP image analysis is shown

Figure 2 shows the CNN architecture designed for image analysis, consisting of convolutional, pooling, dropout, and dense layers. Two convolutional layers with kernel size of 32 and respective filter sizes of 3 × 3 and 5 × 5 were used to extract key features from MMP images. The convolutional layers were followed by a pooling, dropout, and dense layer. Max-pooling was used as pooling layer to compute the maximum value in each patch of each convolved feature map. A dropout layer was added to avoid overfitting. After ‘flattening’ the weights, the softmax function was applied to normalize learned weights and yield a probability distribution. CNN layers were implemented using TensorFlow (version 2.2.0) [23] and Keras (version 2.2.4) [24].

### Performance measures

CNN models were trained to systematically distinguish between MMP-cliffs and corresponding non-AC MMPs. The classification performance of CNN models was evaluated using receiver-operator characteristic (ROC) curves and the area under the ROC curve (AUC). In addition, model performance was assessed with four performance measures including overall accuracy (A), balanced accuracy (BA), weighted mean F1 score [25], and Mathews correlation coefficient (MCC) [26], defined as:

$$MCC=\frac{TP \times TN - FP \times FN }{\sqrt{(TP+FP)(TP+FN)(TN+FP)(TN+FN)}}$$

$$F1=2 \times \frac{TP}{2 TP+FP+FN}$$

$$ROC AUC=\frac{1}{2}- \frac{1}{2} \frac{FP}{FP+TN}+ \frac{1}{2} \frac{TP}{TP+FN}$$

$$A= \frac{TP+FN}{TP+TN+FP+FN}$$

$$BA=\frac{\left(\frac{TP}{TP+FN}\right)+\left(\frac{TN}{TN+FP}\right)}{2}$$

TP, TN, FP, and FN denote true positives, true negatives, false positives, and false negatives respectively.

### Convolutional layer feature visualization

Spatial information from the convolutional layers of trained models was extracted using the Grad-Cam algorithm [17]. Channel-based mean values of the resulting convolutional feature map activation weights were mapped to the original image for feature visualization.

## Results and discussion

### Convolutional neural network models

CNN models were derived to distinguish between MMP-cliffs and non-AC MMPs on the basis of molecular images generated for three distinct activity classes including thrombin inhibitors (target/activity class ID 204), tyrosine kinase Abl inhibitors (class 1862), and mu opioid receptor ligands (class 233). As shown in Fig. 1, MMP images for CNNs combined the shared core structure with the pair of substituents representing the chemical transformation. Images of the three structures constituting an MMP were concatenated horizontally to obtain a single image. In contrast to displaying two compounds forming an MMP side-by-side, this image format contained no redundant substructure (duplicated core).

**Fig. 3:**
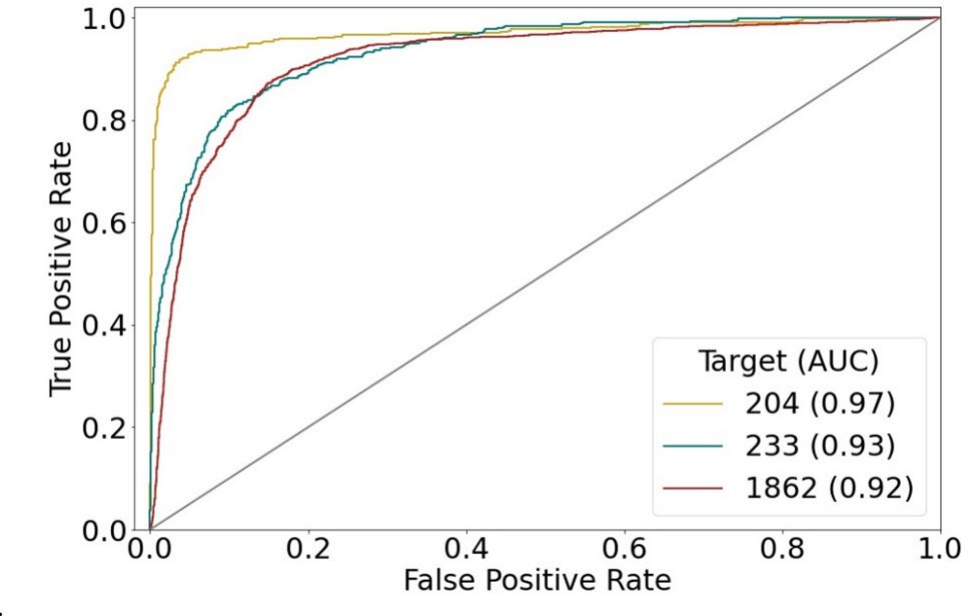
ROC curves. The performance of the best CNN prediction models is monitored in ROC curves. For each curve (activity class, indicated by target ID), the resulting AUC is reported

CNN models were separately trained in 10 independent trials on a set of 4050–10,178 images, dependent on the activity class. Training images were obtained by randomly selecting half of the MMP-cliffs per class (228–561; Table 1) and half of the non-AC MMPs (1797–4856). The resulting models were then tested on the remaining half of the MMP-cliff and non-AC MMP images. ROC curves for the best performing individual classification models are shown in Fig. 3. These CNN models yielded accurate AC predictions, with ROC-AUC values of 0.97 (204), 0.93 (233), and 0.92 (1862). In addition, Table 2 reports the mean prediction accuracy of the CNN models for each activity class on the basis of alternative performance measures. Although training and test sets were imbalanced, i.e., they containing many more non-AC MMPs than MMP-cliffs, the predictions were generally stable (i.e., yielding very low standard deviations) and consistently successful on the basis of all performance measures. Overall, CNN classification accuracy was highest for thrombin inhibitors (class 204), with mean AUC of 0.97 (AUC = 0.97), F1 = 0.85, MCC = 0.83, A = 0.97, and BA = 0.90, followed by class 1862 and 233. Although AUC and A values were also high for class 233 (0.92 and 0.96, respectively), predictions for this class yielded lowest F1 = 0.36, MCC = 0.39, and BA = 0.63 values, indicating that the majority class (non-AC MMPs) was predicted here more accurately than the minority class (MMP-cliffs). In this case, only < 5% of all MMPs represented MMP-cliffs. Thus, these results were expected. The use of balanced training sets would likely further increase prediction accuracy, which is meaningful from a machine learning perspective. However, for AC predictions, balancing MMP-cliff and non-AC MMP training sets would represent an unrealistic scenario because ACs are generally rare among qualifying compound pairs [8]. Regardless, even in the presence of class label imbalance, image-based classification of MMP-cliffs vs. non-AC MMPs was overall surprisingly accurate, more so than we anticipated.

**Table 2:** Mean prediction accuracy

Target	AUC	F1	MCC	Accuracy	
				A	BA
204	0.97 ± 0	0.85 ± 0.02	0.83 ± 0.02	0.97 ± 0	0.90 ± 0.02
1862	0.92 ± 0.01	0.54 ± 0.08	0.50 ± 0.07	0.88 ± 0.01	0.70 ± 0.05
233	0.92 ± 0.02	0.36 ± 0.10	0.39 ± 0.08	0.96 ± 0	0.63 ± 0.05

For MMP-cliff/non-AC MMP classification models, the mean AUC, F1, MCC, global accuracy (A) and balanced accuracy (BA) values ± standard deviations over 10 independent trials are reported

### Image feature visualization

**Fig. 4:**
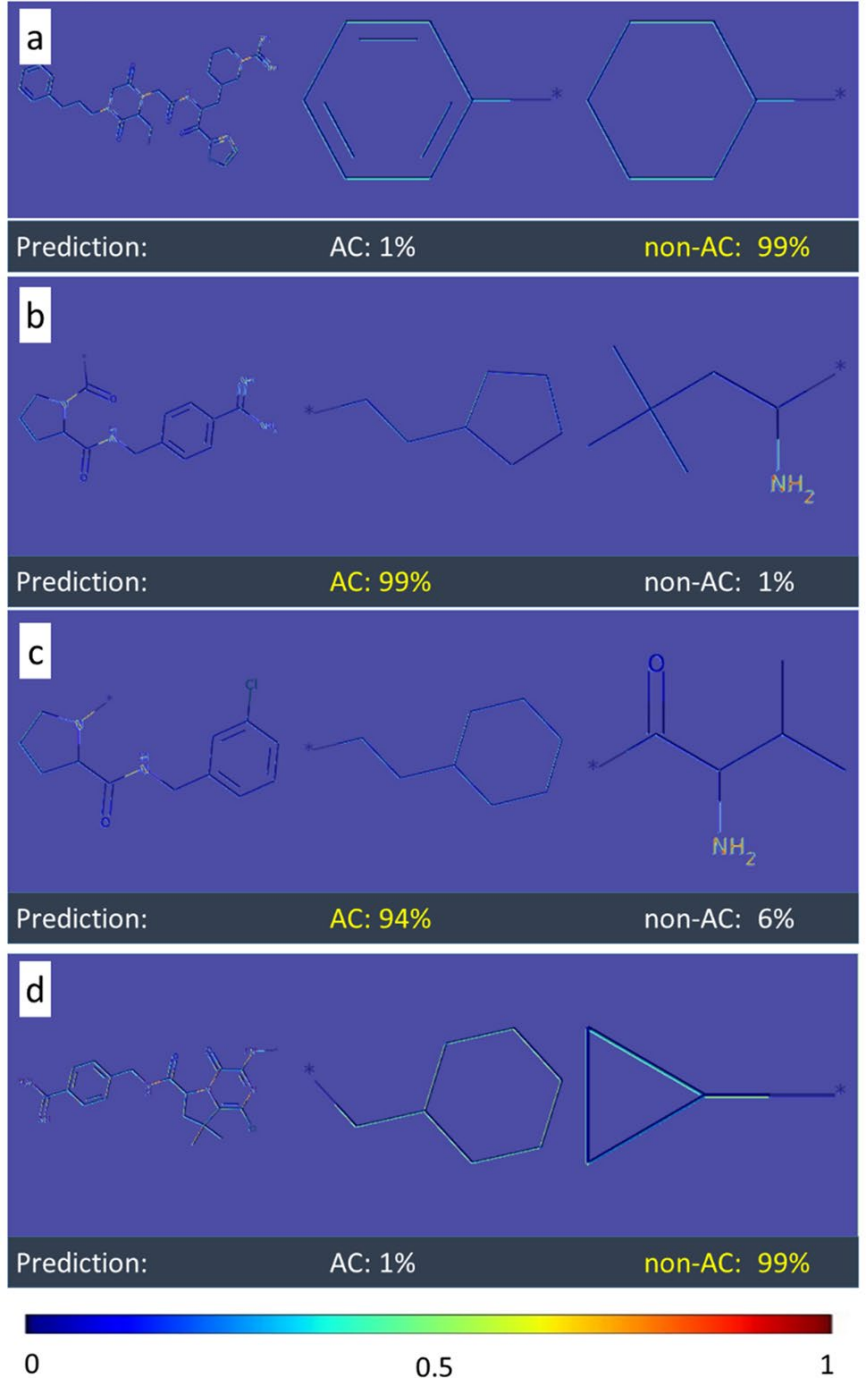
Mapping of activation weights. For four exemplary MMPs from class 204, mean gradient weights of the first convolutional layer are displayed on the respective structures and color-coded according to the given continuous color spectrum. Classification probabilities for each class (AC, non-AC) are given (%) and the correct class label of each MMP is colored in yellow. Shown are **a** a non-AC MMP with phenyl and cyclohexyl substituents, **b** and **c** MMP-cliffs with similar core structures and substituents, and **d** a non-AC MMP with different aliphatic ring substituents

**Fig. 5:**
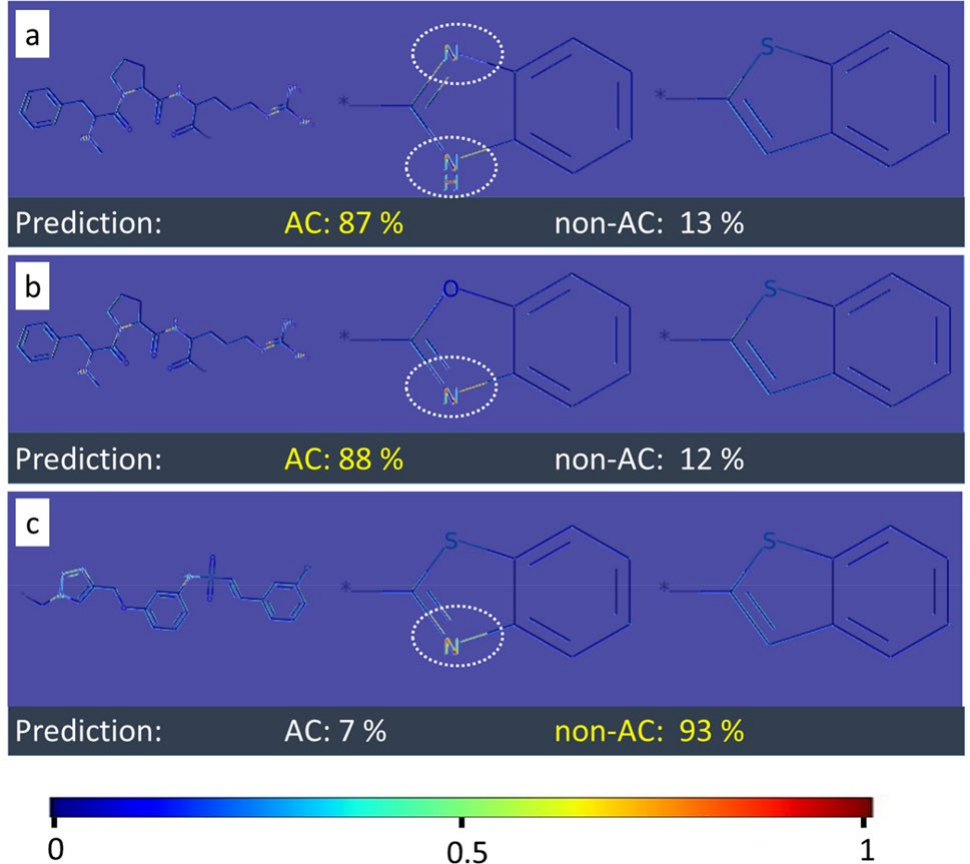
Mapping of activation weights. For three similar MMPs from class 204, mean gradient weights of the first convolutional layer are displayed. The representation is according to Fig. 4. **a** and **b** show MMP-cliffs and **c** shows a non-AC MMP. Highly weighted secondary or tertiary amines are encircled

**Fig. 6:**
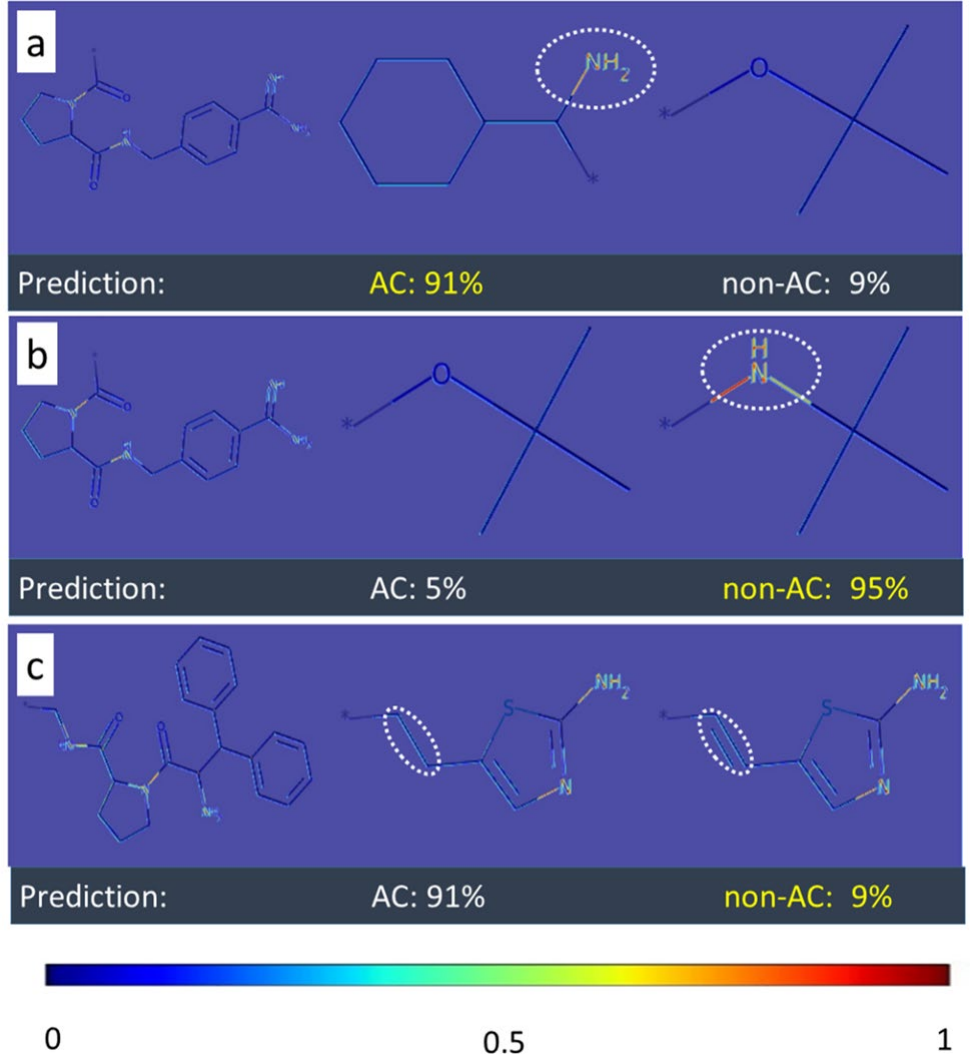
Mapping of activation weights. For three MMPs from class 204, mean gradient weights of the first convolutional layer are displayed. The representation is according to Fig. 4. **a** and **b** show a correctly predicted MMP-cliff and non-AC MMP, respectively. Highly weighted primary or secondary amines are encircled. **c** shows a false positive MMP-cliff prediction. Highly weighted primary and secondary amines are shared by the substituents. The distinguishing single and double bonds were detected with medium weights and are encircled

**Fig. 7:**
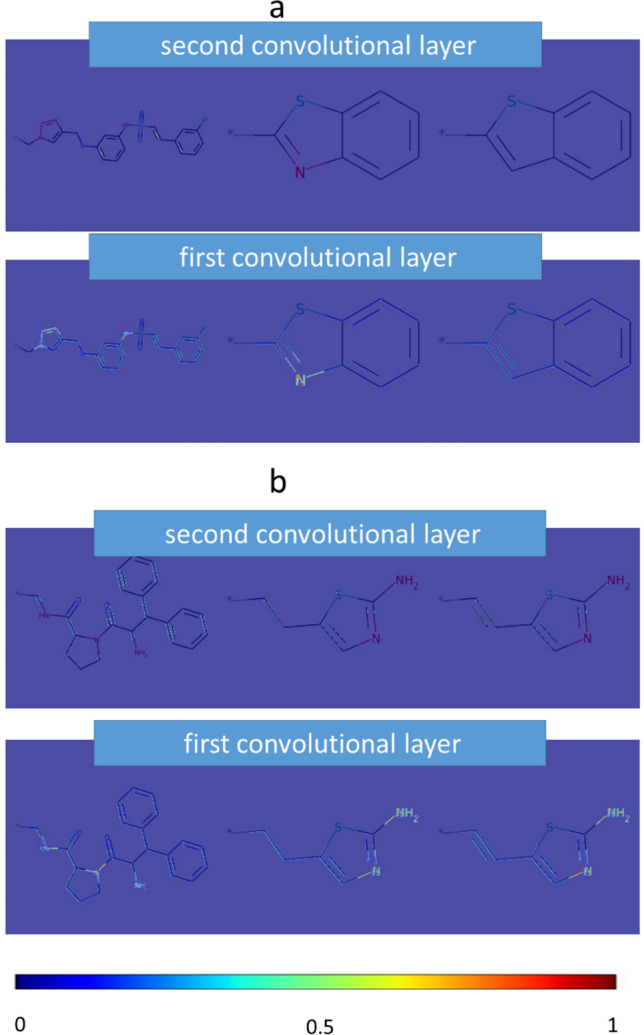
Mapping of first and second layer activation weights. **a** and **b** show the MMPs from Figs. 5c and 6c, respectively, with mean gradient weights of the first and second convolutional layer. Here, weights from the second convolution layer reinforced structural features detected by first layer weights and also identified additional features

Convolutional features naturally retain spatial information, which is lost in fully-connected layers. Therefore, the Grad-Cam algorithm was applied to visualize convolutional layer activation weights [17]. Figures 4, 5 and 6 show examples of original images onto which channel-based mean values of activation weights of the corresponding convolutional feature map were superimposed. All MMPs shown in Figs. 4 and 5 were correctly predicted while Fig. 6 also shows a false positive prediction. Visualization of convolutional layers revealed that most of the key image features were captured by the first convolutional layer. However, in a number of instances, the second convolutional layer was also capable of extracting and emphasizing key features, as shown in Fig. 7. Accordingly, addition of the second convolution layer typically further improved classification accuracy.

### Learning structural features from compound images

The convolutional layer weights of the best performing CNN model (class 204) for MMP images from the test set were systematically extracted and visualized. A compelling observation was that weights from the CNN models detected specific structural features in MMP images. For example, convolutional layers were capable of recognizing primary, secondary, and tertiary amines as well as various ring structures. Moreover, the model was able to differentiate between substituents with different structures. In Fig. 4, the model distinguished between ring and aliphatic substituents, which is clearly evident by comparing mapped convolutional layer weights. Different weight distributions led to accurate predictions of MMP-cliffs and non-AC MMPs with very high probabilities of at least 94%. Furthermore, the model learned to differentiate between alternative cyclic structures, hence accounting for molecular topology.

In Fig. 5, the CNN model assigned high weights to secondary and tertiary amines in rings of substituents of correctly predicted MMP-cliffs and non-AC MMPs. Notably, the presence of different amines was a characteristic feature of all MMPs originating from class 204. However, by comparing the MMP-cliff and non-AC MMP in Fig. 5b and c, respectively, it becomes clear that detecting a tertiary amine alone was not sufficient to distinguish between the MMP-cliff and non-AC MMP because this feature was shared by both. In this case, the core structures of these MMPs were distinct and different core features were detected and assigned high weights, hence illustrating core contributions to accurate predictions. In the MMPs shown in Fig. 6a and b, primary and secondary amines were also detected as distinguishing features in aliphatic substructures. Furthermore, Fig. 6c reports a false positive MMP-cliff prediction. On the basis of the MMP alone, this prediction error cannot be rationalized. To these ends, weights in similar MMPs with different class labels must be compared, as illustrated in Fig. 5. Nonetheless, this example was interesting because the replacement of a single bond with a double bond, i.e., a change in bond order representing a minute chemical modification at the level of images, was detected with medium weights as a distinguishing substituent feature.

Taken together, these convolutional layer weight-based visualizations demonstrated the capacity of the CNN model to detect signature features of compounds from a given activity class (such as the presence of various amines) as well as specific chemical features that distinguished cores and/or substituents of MMPs, including different ring structures, individual functional groups, or bond orders. Mapping weights from different convolutional layers often further emphasized such features or identified additional ones, as illustrated in Fig. 7. The correct detection of specific features distinguishing MMPs with different class labels provided a rationale for the overall accuracy of the AC predictions. Differences between substituents detected by the CNN model can be analyzed at the level of individual MMP images, while understanding differently weighted core features requires comparisons of multiple MMPs. Visualization of key features in MMP cores and substituents aids in the interpretation of CNN model decisions that typically have black box character, hence improving model accessibility.

## Conclusion

In the work, we have attempted the prediction of MMP-cliffs, which are an intuitive AC representation, on the basis of MMP image data using CNN models. To our knowledge, these are the first molecular image-based property predictions at the level of compound pairs. In our proof-of-concept investigation, encouraging accuracy was achieved in systematically distinguishing between MMP-cliffs and non-AC MMPs. While ACs were successfully predicted before using other machine learning approaches, we have been particularly interested in the question whether CNNs are capable of extracting chemical features and small feature differences from images of pairs of structural analogs that correctly distinguish between SAR continuity (embodied by non-ACs) and discontinuity (ACs). Mapping of convolutional layer weights to test compounds and visualizing corresponding structural features put the analysis on a level beyond statistical assessment of prediction accuracy. Visualization revealed the ability of CNN models to detect specific chemical features including distinct substructures and individual functional groups that distinguished structural analogs or MMPs with different properties. Thus, the models were capable to learn chemistry from MMP images, which resulted in successful AC predictions.
